# Maintenance Therapy Can Improve the Oncologic Prognosis and Obstetrical Outcome of Patients With Atypical Endometrial Hyperplasia and Endometrial Cancer After Fertility-Preserving Treatment: A Multicenter Retrospective Study

**DOI:** 10.3389/fonc.2021.808881

**Published:** 2021-12-17

**Authors:** Yijiao He, Jianliu Wang, Yiqin Wang, Rong Zhou, Qun Lu, Guoli Liu, Huiru Tang, Hongyan Guo, Mian He, Guizhu Wu

**Affiliations:** ^1^ Department of Obstetrics and Gynecology, Peking University People’s Hospital, Beijing, China; ^2^ Department of Obstetrics and Gynecology, Peking University Shenzhen Hospital, Shenzhen, China; ^3^ Department of Obstetrics and Gynecology, Peking University Third Hospital, Beijing, China; ^4^ Department of Obstetrics and Gynecology, the First Affiliated Hospital of Sun Yat-sen University, Guangzhou, China; ^5^ Department of Obstetrics and Gynecology, Shanghai First Maternity and Infant Hospital, Shanghai, China; ^6^ Department of Obstetrics and Gynecology, the First Affiliated Hospital of Fujian Medical University, Fuzhou, China

**Keywords:** endometrial cancer, atypical endometrial hyperplasia, fertility-preserving, maintenance therapy, pregnancy, progestogens

## Abstract

**Objective:**

To evaluate the effect of maintenance therapy for patients with atypical endometrial hyperplasia (AEH) and early endometrial cancer (EC) after successful fertility-preserving management on prognosis and pregnancy outcome.

**Methods:**

We performed a retrospectively analysis of 109 young women with atypical endometrial hyperplasia and early endometrioid endometrial cancer who had received complete response after fertility-preserving treatment at 5centers between May 2005 and March 2021. Maintenance therapy regimes included low-dose oral progesterone, levonorgestrel intrauterine device(LNG-IUD) and combination oral contraceptive (COC). The patients were divided into two groups, maintenance therapy group and non-maintenance therapy group. Clinical characteristics, treatment regimens, prognosis, and pregnancy outcome were compared between the two groups.

**Results:**

The overall disease recurrence rate of the maintenance therapy group was significantly lower than that of the non-maintenance therapy group (*P* < 0.001). The recurrence rate of atypical endometrial hyperplasia and endometrial cancer in the maintenance therapy group were significantly lower than those in the non-maintenance group (*P* < 0.001). Maintenance therapy can reduce pregnancy rates and live birth rates. Maintenance therapy can protect the endometrium in patients treated with assisted reproductive technology (ART), greatly reducing the recurrence rate after ART (*P*<0.001).

**Conclusion:**

Maintenance therapy plays a very important protective role in fertility-preserving treatment for patients with atypical endometrial hyperplasia and endometrial cancer, which could significantly reduce the risk of recurrence. It is recommended that patients could receive maintenance therapy as long as possible during the period from achieving complete response to pregnancy preparation if possible. It may provide recurrence-free survival long enough for childless young women to prepare for pregnancy in the future. It can also protect the endometrium of those who are preparing to use assisted reproductive technology, possibly by reducing the risk of recurrence by excessive stimulation with assisted reproductive drugs.

## Introduction

Endometrial cancer (EC) is the most common gynecologic malignancy in developed countries (1). Although EC is typically experienced by postmenopausal women, 3-14% of women are diagnosed equal to or under the age of 40 (2). Atypical endometrial hyperplasia (AEH) is thought to be a precursor of EC, and approximately 25% of AEH will progress to EC if left untreated (3). Currently, the clinical efficacy of fertility-preserving treatment for AEH and endometrioid endometrial cancer (EEC)has been confirmed. Complete response rates with oral high-dose progesterone treatment have achieved 60–98%. However, the recurrence rates are still relatively high (20–50%) (4–6) according to the literature. The median time from achieving complete response to the first recurrence is 14 months (7). This predicts that many patients may face recurrence if they fail to conceive within the median time to recurrence. For patients with fertility demands, the time expected for pregnancy is relatively short. Hence, how to prolong the relapse-free survival to facilitate subsequent pregnancy has become the focus of researchers. Maintenance therapy refers to the continuous administration of low-dose progesterone to protect the endometrium after patients with atypical endometrial hyperplasia and endometrial cancer have achieved complete response through fertility-preserving treatment. The maintenance therapy is aimed at maintaining a disease-free state as long as possible and prolong the fertility preservation duration. The study is intended to explore the clinical significance of maintenance therapy after fertility-preserving treatment for AEH and EC, providing references for standardization and optimization of fertility-preserving treatment for EC.

## Materials and Methods

### Data Source and Patient Selection

The clinical data of 109 patients with AEH and EC who had achieved complete response after fertility-preserving treatment in 5-centers from December 2005 to March 2021 were collected, and the follow-up deadline was June 2021. All patients received standardized evaluation and treatment. The inclusion criteria for fertility-preserving treatment included: histologically proven AEH or grade 1 EEC without myometrial invasion; no signs of suspicious extrauterine involvement by image study; younger than 45 years old; strong willingness to preserve fertility. Written informed consents were obtained from all patients prior to initiating treatment. Patients achieving complete response were assessed by at least two pathologists specializing in gynecological oncology, and all patients were evaluated by pelvic examination, ultrasound scan, and pelvic computed tomography, pelvic magnetic resonance imaging to confirm no tumor in the myometrium and no evidence of lymph node involvement or extrauterine metastasis.

Patients were divided into two groups according to whether maintenance therapy was given after they achieved complete response: 72 cases for Group 1 (maintenance therapy group) and 37 cases for Group 2 (non-maintenance therapy group). This retrospective study was approved by the Independent Ethics Committee (IEC) of Peking University People’s Hospital (approval number: 2016PHB054-01).

### Maintenance Treatment Regimen and Efficacy Evaluation

1. Oral administration of low-dose progesterone (dydrogesterone 20–40 mg/d or progesterone capsules 100–200 mg/d, continuous medication in the second half of the menstrual cycle, duration ≥ 10 days);
2. Levonorgestrel-releasing intrauterine system (LNG-IUS);
3. combination oral contraceptive (COC).

Efficacy Evaluation: (1) stable disease (SD): the pathology was consistent with that prior to treatment, no obvious changes; (2) recurrence: after patients achieved complete response, pathologically confirmed evidence of recurred AEH or EC was noted during the follow-up.

### Follow-Up

After achieving complete response, the patients shall receive transvaginal color doppler ultrasound or pelvic MRI every 3 to 6 months, and hysteroscopic endometrial biopsy shall be performed when the examination suggests abnormal endometrial thickening or unexplained persistent vaginal bleeding.

### Statistical Analysis

In the study, in order to compare the differences between two groups. Fisher’s exact test and chi-square test were performed for categorical variables. Data were described as median values with ranges or as counts with percentages; student t test would be adopted if the value was normally distributed. Kaplan-Meier analysis was adopted to analyze relapse-free survival (RFS), and influencing factors were analyzed by Cox regression analysis. *P* < 0.05 was considered statistically significant. All statistical analyses were performed with IBM SPSS for Windows(version 25.0; IBM Corp, Armonk, NY, USA).

## Results

### Clinical Characteristics

There were 109 patients who achieved complete response after initial treatment, among which, 61 patients were AEH and 48 patients had EC. The mean age of all patients was 31.0 ± 5.0 years. The mean BMI was 26.6 ± 5.0 kg/m^2^. The mean time from initial treatment to complete response was 6.57 ± 5.00 months. The mean follow-up time was 67.87 ± 33.71 months with no deaths. The general characteristics of patients were not significantly different between Group1 and Group 2 (**Table 1**).

**Table 1:** General conditions of patients with atypical endometrial hyperplasia and endometrial carcinoma who achieved complete response (%).

	Total (n = 109)	Group 1 (n = 72)	Group 2 (n =37)	*P* value
Age (years)	31 ± 5	31 ± 5	31 ± 5	0.312
< 30	46 (42.2)	34 (47.2)	12 (32.4)	
30 ≤ and > 35	37 (34)	23 (32.0)	14 (37.9)	
≤ 35	26 (23.8)	15 (20.8)	11 (29.7)	
BMI (kg/m^2^)	26.6 ± 5.0	27.1 ± 5.2	25.6 ± 4.3	0.487
< 25	48 (44.04)	30 (41.7)	18 (48.6)	
≥ 25	61 (55.96)	42 (58.3)	19 (51.4)	
Pathology				0.905
AEH	61 (56.0)	40 (55.6)	21 (56.8)	
EC	48 (44.0)	32 (44.4)	16 (43.2)	
Comorbidity				0.524
N/A	34 (31.2)	21 (29.2)	13 (35.1)	
Yes	75 (68.8)	51 (70.8)	24 (64.9)	
Infertility				0.444
N/A	73 (67.0)	50 (69.4)	23 (62.2)	
Yes	36 (33.0)	22 (30.6)	14 (37.8)	
IR				0.172
N/A	70 (64.2)	43 (59.7)	27 (73.0)	
Yes	39 (35.8)	29 (40.3)	10 (27.0)	
PCOS				0.246
N/A	81 (74.3)	51 (70.8)	30 (81.1)	
Yes	28 (25.7)	21 (29.1)	7 (18.9)	
Initial treatment drug				0.457
Progesterone	93 (85.3)	61 (84.7)	32 (86.5)	
GnRH-a	6 (5.5)	3 (4.2)	3 (8.1)	
Combination therapy(progesterone+ GnRH-a)	10 (9.2)	8 (11.1)	2 (5.4)	
Complete response duration (month)	6.57 ± 5.00	6.49 ± 3.67	6.81 ± 6.96	0.792
Follow-up time (month)	67.87 ± 33.71	59.38 ± 28.73	84.41 ± 36.83	0.001

Group 1, maintenance therapy group; Group 2, non-maintenance therapy group; AEH, atypical endometrial hyperplasia; EC, endometrial cancer; BMI, body mass index; GnRH-a, gonadotropin releasing hormone receptor agonist; IR, Insulin Resistance; PCOS, polycystic ovary syndrome; N/A, Not applicable.

### Comparison of Recurrence Between Different Groups

**Figure 1:**
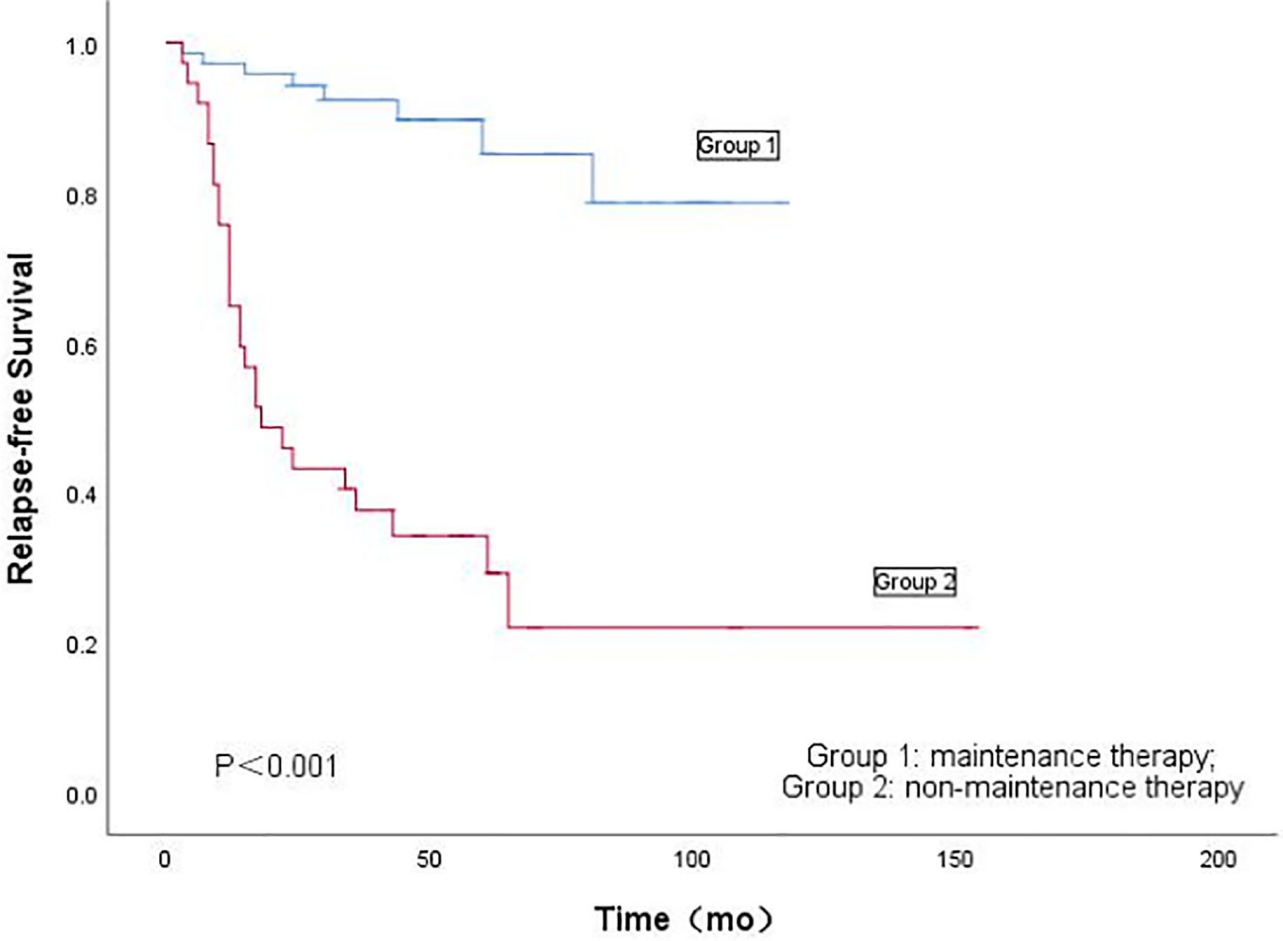
Kaplan-Meier’s analysis of the effect of maintenance therapy on recurrence of fertility-preserving treatment for patients with atypical endometrial hyperplasia and endometrial cancer.

There were 34 patients (31.2%) who had recurrence, as shown in **Table 2**. The recurrence rate of Group 1 is significantly lower than that of Group 2 (11.1% vs. 70.3%; *P* < 0.001). The median recurrence time of the two groups was close (15 months vs. 13 months; *P* = 0.314). Relapse-free survival of AEH and EC is shown in **Figure 1**.

**Table 2:** Recurrence comparison of Group 1 and Group 2 (%).

	Total (n = 109)	Group 1 (n = 72)	Group 2 (n = 37)	*P* value	OR	95% CI
Recurrence	34 (31.2)	8 (11.1)	26 (70.3)	< 0.001	0.11	0.049~0.244
Time from CR to Recurrence (month)	14 (3, 81)	15(3,81)	13 (3,65)	0.314		

CR, complete response.

**Figure 2:**
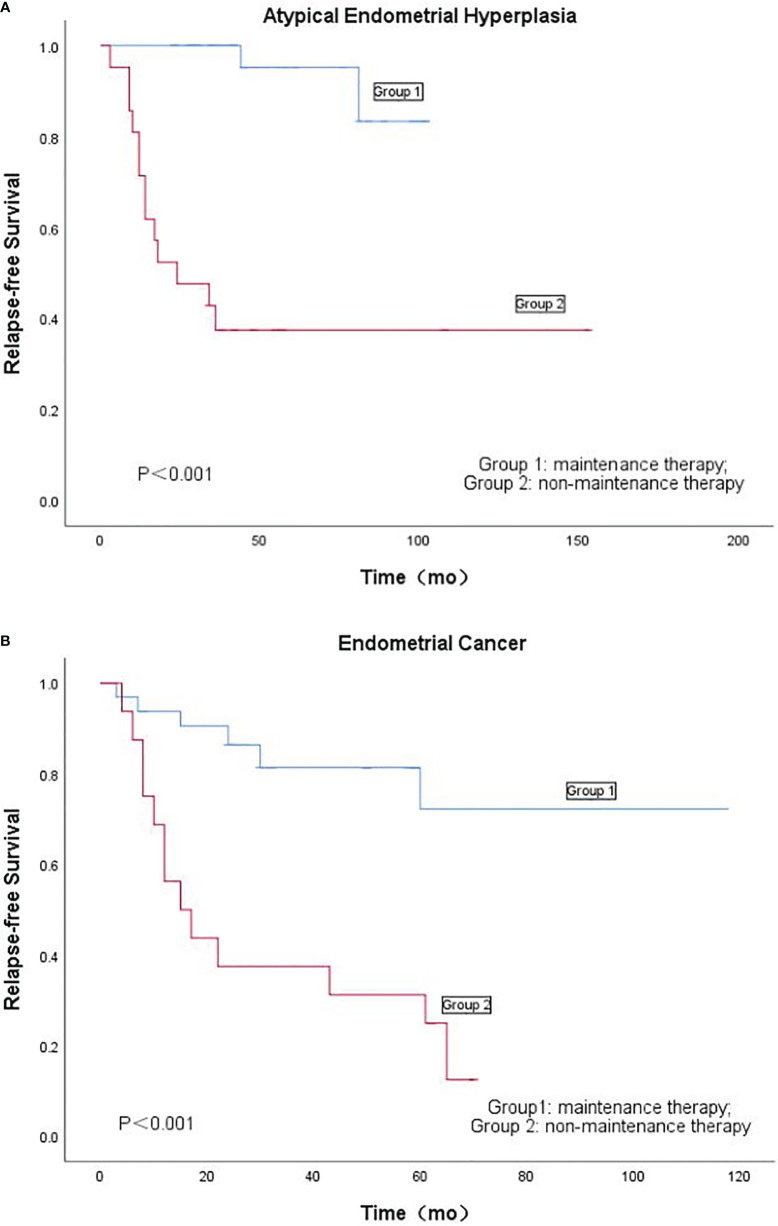
**(A)**: Kaplan-Meier’s analysis of the effect of maintenance therapy on recurrence of patients with atypical endometrial hyperplasia. **(B)** Kaplan-Meier's analysis of the effect of maintenance therapy on recurrence of patients with endometrial cancer.

According to **Table 3**, the recurrence rate of EC was higher than that of AEH, but was not statistically significant (*P* = 0.093). In patients with AEH, the recurrence rate of Group 1 was significantly lower than that of Group 2 (5.0% vs. 61.9%; *P* < 0.001). Kaplan-Meieranalysis of the effect of maintenance therapy on recurrence of patients with AEH is shown in **Figure 2A**. There were no significant differences in time-to-recurrence between the two groups (62.50 ± 26.26 months vs. 16.31 ± 9.72 months; *P*=0.236). In patients with EC, the recurrence rate of Group 1 was significantly lower than that of Group 2 (18.8% vs. 81.2%; *P* < 0.001). No significant differences in time-to-recurrence was observed between the two groups (19.83 ± 22.01 months vs. 21.77 ± 20.82 months, *P* = 0.855). Kaplan-Meier analysis of the effect of maintenance therapy on recurrence of patients with AEH is shown in **Figure 2B**.

**Table 3:** Recurrence comparison of atypical endometrial hyperplasia and endometrial cancer (%).

	Total (n = 109)	AEH (n = 61)	EC (n = 48)	*P* value
		Group 1 (n =40); Group 2 (n =21); *P* value	Group 1 (n =32); Group 2 (n = 16); *P* value	
Recurrence	34 (31.2)	15 (24.6)	19 (39.6)	0.093
		2 (5.0); 13 (61.9); < 0.001	6 (18.8); 13 (81.2); <0.001	
Time from CR to recurrence (month)	21.48 ± 20.25	22.47 ± 19.85	21.16 ± 20.62	0.804
		62.50 ± 26.16; 16.31 ± 9.72; 0.236	19.83 ± 22.01; 21.77 ± 20.82; 0.855	

AEH, atypical endometrial hyperplasia; EC, endometrial cancer; CR, complete response.

### Efficacy of Different Maintenance Regimens

After achieving complete response, there were 72 patients receiving maintenance therapy. There were 49 patients treated with low-dose oral progesterone (dydrogesterone or progesterone capsules), 20 patients with LNG-IUS, and 3 patients with compound oral contraceptive, as shown in **Table 4**. There were 6 patients treated with low-dose progesterone and2 patients with LNG-IUS, who developed recurrence, and no recurrence was observed in patients treated with compound oral contraceptive (*P*=0.793). The time from complete response to recurrence in patients treated with LNG-IUS was longer than that in those treated with low-dose progesterone (42.0 ± 55.2 vs. 26.7 ± 22.2, *P*=0.762). There were no significant differences in pregnancy rates among patients receiving the three maintenance therapy (*P*=0.085).

**Table 4:** Recurrence rate of patients with different maintenance therapy regime after achieving complete response.

	Total (n = 72)	Low-dose Progesterone (n = 49)	ING-IUS (n = 20)	COC (n = 3)	*P* value
Maintenance therapy duration (m)	21.7 ± 24.3	22.7 ± 24.7	21.6 ± 25.5	4.7 ± 2.9	0.866
Recurrence(%)	8 (11.1)	6 (12.2)	2 (10)	0 (0)	0.793
Time from CR to recurrence (m)	30.5 ± 28.9	26.7 ± 22.2	42.0 ± 55.2	–	0.762
Desire to pregnancy (%)	41 (56.9)	34 (69.4)	5 (25.0)	2 (66.7)	0.003
Pregnancy(%)	19 (46.3)	18 (52.9)	0 (0)	1 (50)	0.085

ING-IUS, levonorgestrel intrauterine sustained release system; COC, combination oral contraceptive; CR, complete response.

**Figure 3:**
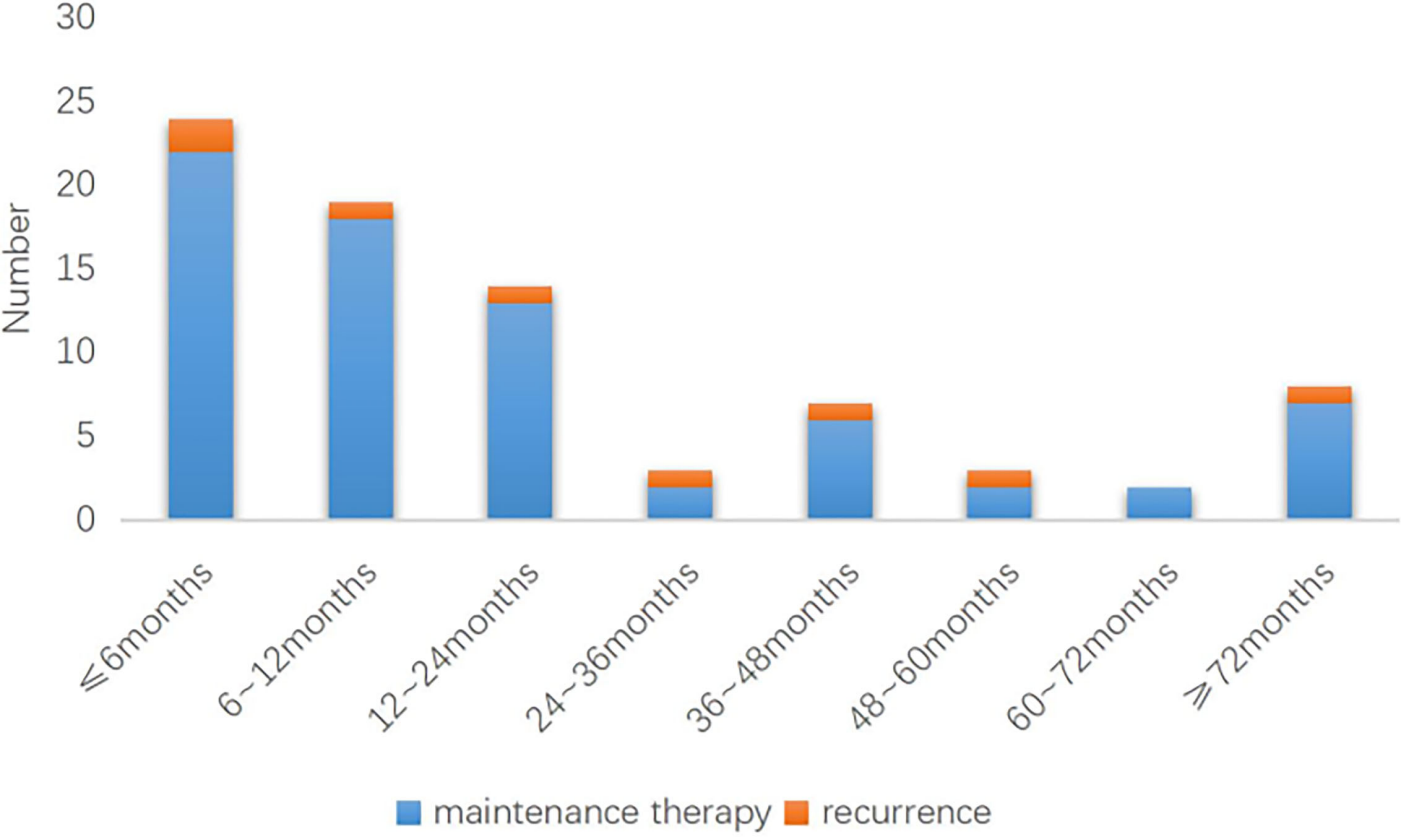
Recurrences occurred at different period in group 2 (n=26).

The length of maintenance therapy varies dependng on how long it takes each patient to start trying to conceive. In Group 1, the duration of maintenance therapy was 21.7 ± 24.3 months. The duration of maintenance therapy was divided into eight segments, as shown in **Figure 3**. In Group 1, 2 of 22 patients with ≤6 months of maintenance therapy experienced recurrence; recurrence occurred in 1 of the 18 patients whose duration of maintenance ranges from 6 to 12 months, 1 of the 13 patients whose duration of maintenance ranges from 12 to 24 months, 1 of the 2 patients whose duration of maintenance ranges from 24 to 36 months, 1 of the 6 patients whose duration of maintenance ranges from 36 to 48 months, 1 of the 2 patients whose duration of maintenance ranges from 48 to 60 months,1 of the 7 patients whose duration of maintenance ranges over 72 months of maintenance therapy. Two patients underwent maintenance therapy for 60 to 72 months without recurrence.

**Figure 4:**
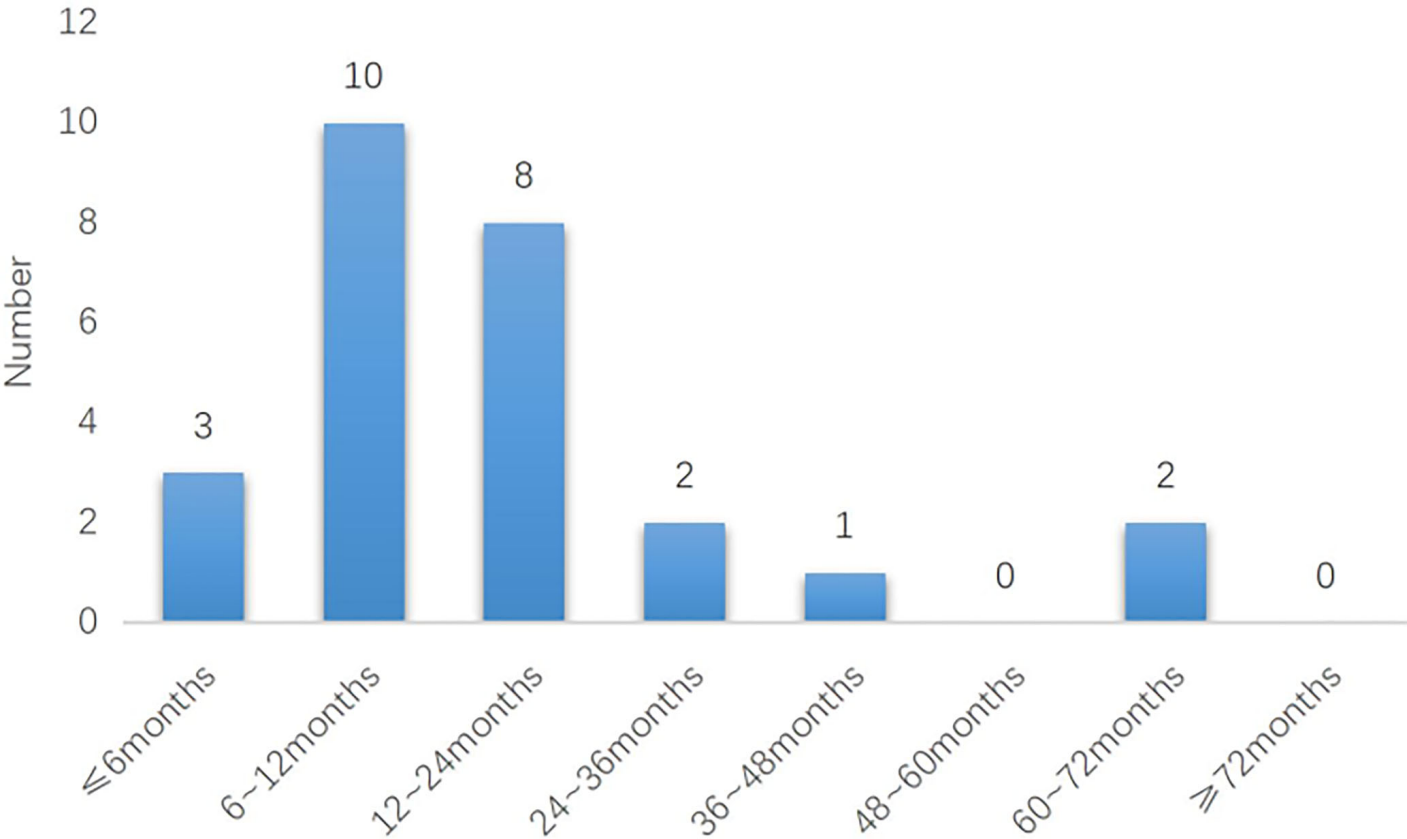
The relationship between maintenance therapy and recurrence in different time periods in The Group 1 (n = 72).

In Group 2, recurrence at different time points since complete response was shown in **Figure 4**. We concluded that in the non-maintenance group, recurrence was concentrated between 6 months and 24months after achieving complete response.

### Pregnancy Outcome

**Figure 5:**
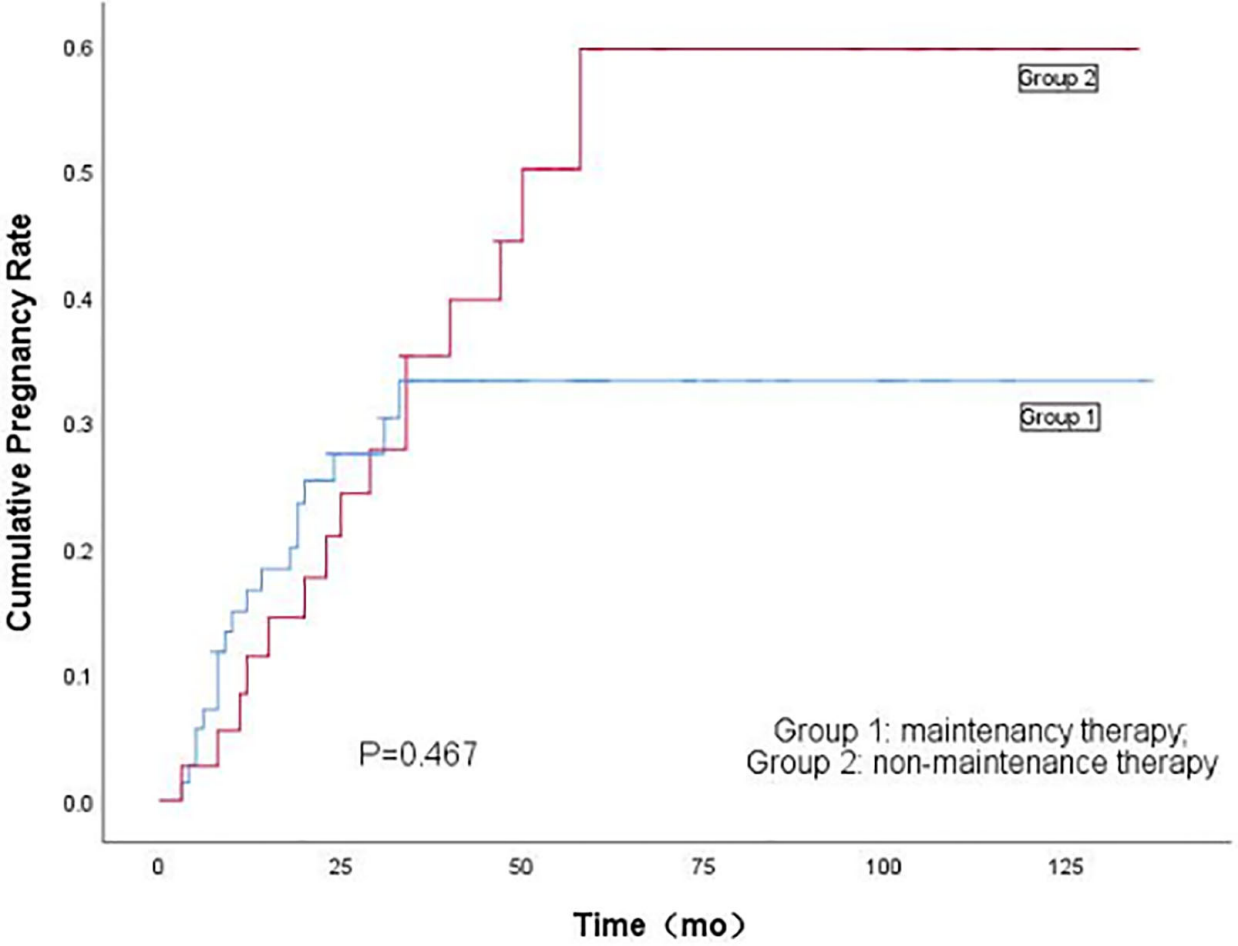
Kaplan-Meier's analysis of the effect of maintenance therapy on cumulative pregnancy rate Between Group 1 and Group 2.

In this study, 70 patients (64.2%) had pregnancy desire among the 109 patients with complete response. There were 34 (48.6%) successful pregnancies. There is no statistical difference in the pregnancy rates between Group 1 and Group 2 (46.3% vs. 51.7%; *P*=0.131). There were 28 (40.0%) full-term pregnancies (eight patients with natural pregnancy and twenty patients with assisted reproductive technology pregnancy). Among them, 12 newborns were delivered vaginally and 16 were delivered by cesarean section. There is no statistical difference in the live birth rates and delivery mode between Group 1 and Group 2 (39.0% vs. 41.3%; *P*=0.248), as shown in **Table 5**. Kaplan-Meier analysis of the impact of maintenance therapy on the cumulative pregnancy rates of Group 1 and Group 2 is shown in **Figure 5**.

**Table 5:** Pregnancy outcomes of patients with different treatment after achieving complete response (%).

	Total (n = 109)	Group 1 (n = 72)	Group 2 (n = 37)	*P* value
Desire for pregnancy	70 (64.2)	41 (56.9)	29 (78.3)	0.027
Expecting natural	20 (28.6)	10 (24.4)	10 (34.5)	0.000
ART	50 (71.4)	31 (75.6)	19 (65.5)	0.000
Pregnancy	34 (48.6)	19 (46.3)	15 (51.7)	0.131
Way of conception:				0.755
Natural conception	10 (29.4)	6 (31.6)	4 (26.7)	0.371
ART	24 (70.6)	13 (68.4)	11 (73.3)	0.273
Live birth	28 (40.0)	16 (39.0)	12 (41.3)	0.248
Way of live birth conception				0.629
Natural conception	8 (28.6)	4 (25.0)	4 (33.3)	0.561
ART	20 (71.4)	12 (75.0)	8 (66.7)	0.822
Delivery mode				0.508
Vaginal	12 (42.9)	6 (37.5)	6 (50.0)	
Cesarean	16 (57.1)	10 (62.5)	6 (50.0)	

AEH, atypical endometrial hyperplasia; EC, endometrial cancer; ART, assisted reproductive technology.

Forty-three patients had undergone assisted reproductive technology after having achieved complete response, fourteen patients (32.5%) had recurrence. The recurrence rate of patients in Group 1 who received assisted reproductive technology was significantly lower than that in Group 2 (9.1% vs. 57.1%; *P*=0.001). The pregnancy rate of patients in Group 1 who received assisted reproductive technology was slightly higher than that in Group 2 (68.2% vs. 42.9%, *P*=0.095), while there was no significant difference in the live birth rate between the two groups (59.1% vs. 33.3%; *P*=0.091), as shown in **Table 6**.

**Table 6:** Effect of assisted reproductive technology on outcomes of atypical endometrial hyperplasia and endometrial cancer after fertility-preserving treatment (%).

	Total (n = 109)	Group 1 (n = 72)	Group 2 (n = 37)	*P* value
ART after achieving CR	43 (39.4)	22 (30.6)	21 (56.8)	0.112
Recurrence after ART	14 (32.5)	2 (9.1)	12 (57.1)	0.001
Pregnancy after ART	24 (55.8)	15 (68.2)	9 (42.9)	0.095
Live birth after ART	20 (46.5)	13 (59.1)	7 (33.3)	0.091

AEH, atypical endometrial hyperplasia; EC, endometrial cancer; CR, complete response; ART, assisted reproductive technology.

## Discussion

According to the expert consensus on the fertility-preserving treatment of EC in China (8), three maintenance therapy regimens are proposed: oral low-dose progesterone in the second half of the menstrual cycle (e.g., oral dydrogesterone 20–40 mg/d for at least 10 continuous days), intrauterine placement of levonorgestrel intrauterine system, and compound oral contraceptive. These three regimens are commonly used for menstrual cycle regulation or contraception in non-cancer patients and are now beginning to have new clinical applications in the maintenance therapy of AEH and EC. In a recent study reported by Park (6), patients received two maintenance therapy regimens: low-dose cyclic progestin or a progestin-containing intrauterine device (IUD). Maintenance therapy started when the patient achieved complete response and the treatment discontinuation time depends on the patient’s condition and when she attempted to get pregnant.

There were no significant differences between Group 1 and Group 2 in age, BMI, pathology type, presence or absence of comorbidities, and complete time of response after initial treatment, indicating that the basic conditions of the two groups of patients were similar. The follow-up time of Group 2 was significantly longer than that of Group 1, because some patients in Group 2 have an earlier onset than patients in Group 1. In fact, most patients who had achieved complete response did not receive any maintenance therapy in China in the years before fertility-preserving treatment began on a large scale.

According to relevant study results, maintenance therapy has brought great benefits to patients with AEH and EC. As reported by Park (9), the maintenance therapy was significantly correlated with low recurrence rate (OR, 0.19; 95% CI, 0.05–0.78; *P*=0.022). In Nomura’s study (10), there were 18 cases of atypical polypoid adenomyoma, all of which were treated with progesterone. the results indicated that none of them progressed during the maintenance therapy, and the maintenance therapy was a significant factor to reduce the hysterectomy rate (HR, 0.098; 95% CI, 0.012–0.78; *P*=0.015). The recurrence rate of Group 2 in this study was significantly higher than that of Group1 (OR: 0.110; 95%CI: 0.049-0.244; *P* < 0.001). These data show a significant positive effect of the maintenance therapy on the oncologic prognosis. Therefore, we suggest that maintenance therapy should be used routinely if the patients are not anxious to get pregnant.

In addition, the recurrence rate of patients with AEH and EC in Group 1 was significantly lower than that in Group 2. The results suggest that maintenance therapy can reduce not only the recurrence rate of AEH but also EC. It could be seen by comparison that the time from complete response to recurrence in Group 1 and Group 2 was close (*P*=0.314), indicating that the duration of maintenance therapy may not have a significant effect on the recurrence time. However, more data from patients with relapses need to be collected to justify this conclusion. In this study, 68.1% of the patients were treated with low-dose progesterone, while only a few patients were treated with levonorgestrel intrauterine system (27.8%) and compound oral contraceptives (4.1%). The results indicated that there are no significant differences in the prognostic influence of the three maintenance therapy regimens, as shown in **Table 4**. LNG-IUS is an economical and convenient treatment option. The clinical significance of COC in fertility-preserving treatment of AEH and EC is worth exploring. Due to the large difference in the number of cases among the three regimens, no significant difference was found. This suggests that we need to expand the sample size in the subsequent study.

Pregnancy rate and live birth rate were slightly lower in Group 1, but the differences were not significant between the two groups. It indicates maintenance therapy does not have significant effect on pregnancy rate and live birth rate. In this study, 71.4% of the patients chose assisted reproductive technology, and the pregnancy rate of patients treated with assisted reproductive technology in Group 1 was close to that of patients in Group 2. Though it has been reported in the literature that assisted reproductive technology does not increase the risk of recurrence or affect the disease-free survival of patients (11), the recurrence rate of patients in Group 2 who received assisted reproductive technology was significantly higher than that of patients in Group 1 (57.1% vs. 9.1%; *P*=0.001), indicating that maintenance therapy could protect the endometrium when assisted reproductive technology was being applied, thereby reducing the risk of recurrence.

The addition of low-dose progesterone in the second half of the menstrual cycle and compound oral contraceptives could not only regulate the menstrual cycle, but also protect the endometrium. In the relevant literature, the concept of maintenance therapy has not been proposed uniformly, nor has its clinical significance been analyzed in detail. What we can do to reduce the recurrence rate, protect the endometrium, and guarantee the safety of the patients during the indefinite length of time between when the patient reaches complete response and when she successfully becomes pregnant is still a problem. The results of this study provided objective data as a basis for settling it, enabling relevant investigators to have further exploration. However, as a retrospective study, this study is still limited. It aims to arouse the attention of relevant investigators on this issue, and more multicenter prospective study should be conducted to address this issue in the future.

## Conclusions

This study demonstrated that maintenance therapy plays a very important protective role in fertility-preserving treatment for AEH and EC. It is recommended that patients could receive maintenance therapy as long as possible during the period from achieving complete response to pregnancy preparation if possible, because the therapy can reduce the risk of tumor recurrence. It can also protect the endometrium of those who are preparing to use assisted reproductive technology, possibly by reducing the risk of recurrence by excessive stimulation with assisted reproductive drugs.

Moreover, these results have profound effects on fertility-preserving treatment of AEH and EC. The development of treatment strategy in oncology has represented a paradigm shift.

## Data Availability Statement

The original contributions presented in the study are included in the article/supplementary material. Further inquiries can be directed to the corresponding author.

## Ethics Statement

Written informed consent was obtained from the individual(s) for the publication of any potentially identifiable images or data included in this article.

## Author Contributions

YH: Data collection and sorting, data analysis, article writing; JW: Research guidance, paper revision, financial support; YW: Clinical diagnosis and treatment, data collection; RZ: Clinical diagnosis and treatment, research guidance; QL: Clinical diagnosis and treatment, research guidance; GL: Clinical diagnosis and treatment; HT: Data collection; HG: Data collection; MH: Data collection; GW: Data collection.

## Funding

This work was supported by the National Key Technology R&D Program of China (Grant No. 2019YFC1005200 and 2019YFC1005204), And by the Beijing Health Care Promotion Program of Technological Achievements and Appropriate Technology (Grand No. BHTPP202050) and the National Natural Science Foundation of China (No. 82072861).

## Conflict of Interest

The authors declare that the research was conducted in the absence of any commercial or financial relationships that could be construed as a potential conflict of interest.

## Publisher’s Note

All claims expressed in this article are solely those of the authors and do not necessarily represent those of their affiliated organizations, or those of the publisher, the editors and the reviewers. Any product that may be evaluated in this article, or claim that may be made by its manufacturer, is not guaranteed or endorsed by the publisher.
